# Evaluating Two Common Strategies for Research Participant Recruitment Into Autism Studies: Observational Study

**DOI:** 10.2196/16752

**Published:** 2020-09-24

**Authors:** Kelli L Ahmed, Andrea R Simon, Jack R Dempsey, Rodney C Samaco, Robin P Goin-Kochel

**Affiliations:** 1 Baylor College of Medicine Houston, TX United States; 2 Autism Center Texas Children's Hospital Houston, TX United States; 3 Children's Hospital Colorado Aurora, CO United States; 4 University of Colorado School of Medicine Aurora, CO United States

**Keywords:** autism spectrum disorder, participant recruitment, social media, Facebook, radio, genetic studies

## Abstract

**Background:**

Ongoing research is necessary to better understand the causes of autism spectrum disorder (ASD), the developmental outcomes for individuals diagnosed with ASD, and the efficacy of the interventions. However, it is often difficult to recruit sufficient numbers of participants for studies, and despite the prevalence of ASD (currently estimated to affect 1 in 54 children), little research has focused on how to efficiently recruit participants with ASD.

**Objective:**

The aim of this study was to determine the efficacy of two different paid advertisements—social media and radio advertising—in recruiting participants for a study enrolling people with ASD and their family members by examining the number of participants enrolled, the cost per participant, and the geographic reach of each type of advertising.

**Methods:**

We examined participant enrollment in a study following nonoverlapping paid advertisements on a popular FM radio station (aired in three cities across two states) and Facebook (six advertisements that ran in five cities across two states). The total paid investment in the radio campaign was $12,030 and that in the Facebook campaign was $2950. Following the advertising campaigns, 1391 participants in the study who were affiliated with the Houston, Texas, site received email invitations to participate in a brief survey about the ways in which they learned about the study (eg, social media, medical provider, website) and which of these were most influential in their decisions to participate; 374 (26.8%) of the participants completed this survey.

**Results:**

Social media advertising outperformed radio in all three parameters examined by enrolling more participants (338 vs 149), with a lower average cost per participant ($8.73 vs $80.74) and a wider geographic reach, based on a comparison of the number of zip codes within and outside of Texas for questionnaire respondents who rated social media as the most influential method of contact (n=367, χ^2^_1_=5.85, *P*=.02). Of the 374 survey participants, 139 (37.2%) reported that they had seen the study on social media prior to enrollment, while only 9 (2.4%) said they heard about it via radio.

**Conclusions:**

Our findings suggest that advertising on social media can efficiently reach a large pool of potential participants with ASD, increasing the likelihood of meeting study enrollment goals. Researchers should consider allocating at least some portion of recruitment dollars to social media platforms as a means of quickly and inexpensively reaching out to their target populations, including for studies with in-person procedures.

## Introduction

### Background

A genetic contribution to the development of autism spectrum disorder (ASD) has been well established; however, for the majority of individuals with ASD, the genetic contributions remain unknown [1,2]. Continued study of ASD is critical for identifying additional causal mechanisms as well as for developing and improving personalized treatment approaches that facilitate optimal outcomes. However, successful recruitment for such studies can be challenging and can require substantial time and financial investments [3]. Using the National Library of Medicine clinical trials registry, Carlisle et al [4] showed that of 2579 phase 2 and 3 intervention clinical trials that closed in 2011, 19% terminated early because of low enrollment or enrolled less than 85% of their target enrollment numbers. A number of factors may contribute to recruitment challenges, including the demands of the study, the appeal of the advertisement, and importantly, the population being recruited. Understanding these factors and how they affect study enrollment is key to reducing the likelihood of underenrollment. Underenrolled studies consume resources that could be dedicated to other projects. Further, they may lack the statistical power necessary to uncover meaningful results and support the conclusions of the study [5]. Thus, understanding how to recruit participants *effectively* and *efficiently* is of the utmost importance to ensure successful completion of studies and to advance scientific knowledge [6].

Challenges in reaching clinical recruitment goals are ubiquitous; however, this is not due to lack of effort. Research teams have used a variety of recruitment methods to seek participants, with one such strategy being radio advertising. Recruiting for research through radio advertisements has been a popular strategy for decades [7], and its appeal lies in the ability to reach a wide audience with limited effort on behalf of the study team. According to a recent Nielsen report, nearly 92% of Americans are weekly radio listeners, with comparable listening rates across a variety of demographics (eg, sex, age cohort, and racial and ethnic groups [8]). Moreover, more than 93% of radio listeners continue to listen during commercials [9]. However, reports using radio advertising for research recruitment show that it can be costly, ranging from $80 to $827 per enrolled participant [10-14]. In four of these studies, radio advertising was the most expensive recruitment strategy used [11-14]. Additionally, the success of radio advertising in helping to achieve recruitment goals varies; in some published reports, radio advertising helped recruit the most participants of all strategies used [10,11,15], while in another study, only 1 participant (0.2%) was recruited in this way [16].

A modern recruitment strategy that has gained popularity in recent years is advertising on social media platforms, such as Facebook or Instagram. In the United States, 88% of people aged 18 to 29 years and 78% of people aged 30 to 49 years are connected to one or more social media platforms [17]. The most popular of these is Facebook, which is used by 68% of adults in the United States, 74% of whom visit the site daily [17]. Indeed, in a review of 27 studies, advertising on Facebook for research recruitment tended to be more time- and cost-effective than traditional research advertising strategies, with costs ranging from $1.36 to $110 per completed participant [3]. Moreover, Facebook ads can be tailored to target a specific audience. Focusing on families managing ASD, research has shown that these parents often rely heavily on *other* parents of children with ASD for information and support, including via Facebook groups [18-20]. Collectively, this suggests that social media advertising may be a particularly appealing way to reach select audiences and broadly share information about research opportunities, including to parents of children with ASD.

### Purpose

Despite the high prevalence of ASD (1 of every 54 children in the United States) [21], there is a dearth of research that directly addresses how to best recruit participants with ASD and their families into research studies. In fact, to our knowledge, there is only one such report, which focused specifically on recruitment of Hispanic participants with ASD [22]. Thus, there is a great need for additional research that identifies the most effective recruitment strategies for participants with ASD. To this end, we examined two different mechanisms of paid advertising (radio and Facebook) in terms of number of participants recruited, cost, and geographic reach within the context of the SPARK (Simons Foundation Powering Autism Research for Knowledge) project. For the purposes of the current study, we focused on radio and Facebook recruitment strategies at the Houston SPARK site. Additionally, we solicited feedback from Houston-affiliated participants about their recruitment and enrollment experiences to compare against enrollment numbers and reflect participant perceptions about the most influential recruitment strategies.

### SPARK: Project Overview

SPARK is a national, multi-site effort to enroll 50,000 individuals with ASD and their biological family members into a web-based genetic and phenotypic repository [23]. Briefly, more than 20 clinical sites across the United States form a clinical network to recruit potential participants and assist them throughout the enrollment process. SPARK participation is open to any individual living in the United States with a professional diagnosis of ASD or a dependent with a professional diagnosis of ASD. ASD diagnosis is ascertained through self-reporting, which has previously been shown to have >90% reliability [24]. Interested participants visit the study website, create a web-based profile, and complete the web-based enrollment forms to create a “primary” account. Because this “primary” account holder is required to be an independent adult, they can be either a parent or guardian of an individual with ASD or an independent adult with ASD. Once enrolled, each participating member of the family receives an at-home saliva collection kit with instructions on sample collection. Participants subsequently mail completed kits back to the laboratory for DNA analysis. Families can follow this process on their own or can contact a research coordinator for support.

At the Houston SPARK site, multiple strategies were used to recruit participants for SPARK; however, because existing literature suggested that the potential reach, popularity, and success of paid radio and Facebook campaigns may be similar, these two approaches were singled out at the Houston site for further examination. Both campaigns occurred during nonoverlapping months within a 6-month timeframe, during which enrollment numbers were tracked daily through the web-based coordinator portal.

## Methods

### Recruitment Strategies

For the radio outreach, three FM radio campaigns were scheduled across two states (three cities) at a total investment of $12,030. All three campaigns ran during a 4-week period between November 28 and December 26, 2016, on a popular station that played holiday music. The aired advertisement was 30 seconds long and ran a total of 342 times across markets. Interested radio listeners were invited to text the word “SPARK” to a short-code telephone number (555-888) to receive information about a web-based US autism research study. These “subscribers” received three immediate text messages with 1) the study’s institutional review entity–approved call to action, 2) the hyperlinked URL to register on the internet, and 3) the research coordinator’s contact information. Two weeks after texting the short-code, subscribers received an automated reminder message with the enrollment link to the study.

For the social media campaign, a series of six paid Facebook ads were placed across two states (five cities) between March 23 and May 22, 2017, at a total investment of $2950. Five of these campaigns consisted of institutional review board–approved recruitment language, with a 2:07 minute video of the principal investigator at the Houston site explaining the study, while one campaign used the approved call-to-action language and an image to invite participants to enroll in person with the assistance of the study team. The geographic radius, campaign length, and dollar investment for each Facebook campaign varied slightly. However, all six campaigns were targeted using identical audience criteria for adults aged 22 to 55 years with interests in autism awareness organizations or special education. The Facebook ads targeted individuals who previously endorsed interest in the National Autism Association, World Autism Day, National Autistic Society, Special Education, Autism Spectrum Awareness, Stand Up for Autism, Asperger syndrome awareness, Autism Community Network, Autism Awareness, Autism Society of America, or Autism Speaks.

During both paid advertising campaigns, a minimal number of other traditional recruitment methods were used simultaneously. For example, flyers were posted in the clinic before, during, and between the two campaigns. Other “background” recruitment methods included physician referral, word of mouth, and information about the project on the clinic’s website. These efforts were not consistently tracked; however, they remained consistent throughout both the radio and social media campaigns.

### Questionnaire

Study data were collected and managed using Research Electronic Data Capture (REDCap) tools hosted at Texas Children’s Hospital [25]. The questionnaire ascertained information about the participants’ recruitment and enrollment experiences with SPARK, including all the ways they heard about the project before creating their web-based profile and which way was most influential in their decision to enroll. An invitation with a link to the questionnaire was emailed to the primary account holders, followed by three additional email reminders that were each sent one week apart.

### Participants

The sampling pool for the questionnaire consisted of 1391 primary account holders affiliated with the Houston SPARK site who enrolled and consented to providing a saliva sample between April 21, 2016, and February 29, 2018. These participants received an invitation via email to complete a questionnaire about their recruitment and enrollment experiences with SPARK. A total of 374/1391 (26.8%) participants responded (see Table 1). The mean age of the 1391 respondents was 39 years (SD 8.7), 91% (1266/1391) identified as female, and 96% (1335/1391) were the parent or grandparent of a person with ASD, while 11 (3%) reported that they themselves had ASD (mean 30 years, SD 10.1 years). The mean age of the individuals with ASD (n=404, including the 11 independent adults) at the time of survey completion was 9.9 years (SD 6.6 years). On average, the 374 survey respondents reported a total of four people living in their household, with between 2 and 3 people participating in the SPARK study; 38 people (10.2%) reported that more than one person with ASD lived in their home.

### Demographic Information

Race and ethnicity data were gathered for SPARK participants through two sources. For participants who are also affiliated with our clinical site, we obtained race and ethnicity data through our Epic medical record system. Additionally, SPARK distributed a survey in July 2017 to allow families to voluntarily provide race and ethnicity data. The demographic data for the state of Texas were obtained from the US census website [26]. Approved researchers can obtain the SPARK population dataset described in this study by applying at the Simons Institute Autism Research Initiative portal [27].

**Table 1:** Demographics of the survey recipients (N=1391), n (%).

Characteristic		Survey responders (n=374)	Survey nonresponders (n=1017)
**Race**			
	White	243 (65.0)	536 (52.7)
	Black or African American	36 (9.6)	92 (9.0)
	Asian or Pacific Islander	15 (4.0)	35 (3.4)
	Native American or American Indian	2 (0.5)	1 (<0.1)
	More than one race	25 (6.7)	39 (3.8)
	Other	13 (3.5)	17 (1.7)
	Data unavailable	40 (10.7)	297 (29.2)
**Ethnicity**			
	Hispanic	108 (35.2)	252 (35.0)
**Highest level of education**			
	High school diploma, GED^a^, or less	31 (8.3)	N/A^b^
	Some college, no degree	88 (23.5)	N/A
	Associate’s degree	54 (14.4)	N/A
	Bachelor’s degree	101 (27.0)	N/A
	Graduate degree	100 (27.0)	N/A

^a^GED: General Education Development.

^b^N/A: not applicable.

### Data Analysis

To determine the number of participants from the radio campaign, we included any enrollments during the campaign, which lasted one month, as well as during the month following the campaign. This allowed all subscribers to receive a two-week reminder about enrollment and gave them an additional two weeks to enroll. More simply, any participant who enrolled in SPARK from November 28, 2016, through January 31, 2017, was counted for the radio campaign. To keep the amount of time consistent between the radio and Facebook campaign groups, we only counted participants who enrolled during the Facebook campaign itself, which ran for two months, from March 23 to May 22, 2017. Participants from both campaigns were given a deadline of October 30, 2017, to return the saliva kit for the individual with ASD.

To determine the effectiveness of each recruitment campaign, we examined three factors: number of participants, cost, and geographic reach. Two different numbers of participants were examined: the number of individuals with ASD who were enrolled and returned the saliva kit to the lab during the course of each campaign, and the number of primary account holders who reported on the questionnaire that they had heard about the study on radio or social media before registering. The cost per participant was determined by dividing the total cost of the campaign by the number of individuals with ASD who enrolled on the internet during that campaign. Geographic reach was examined using the Mapping toolbox in MATLAB (R2018b, MathWorks) to plot the zip codes provided by participants upon registration by latitude and longitude [23]. Using the zip codes provided by the survey participants, the numbers of zip codes in Texas versus outside of Texas were compared using chi-square analysis for the participants who responded that social media was their most influential method of contact.

## Results

### Number of Participants Recruited

#### Radio and Facebook Campaigns

Across the six-month time period encompassing both the radio and Facebook advertising campaigns, 568 individuals with ASD enrolled in the SPARK project. Of those 568 individuals, 520 (91.5%) also consented to providing a saliva sample, and 295 saliva kits were returned for an individual with ASD (as of October 30, 2017). During the radio advertising campaign, 378 people texted the short code, and 149 individuals with ASD were enrolled in the study; 140 of these individuals (94.0%) consented to providing a saliva sample, and 83 (55.7%) ultimately returned the saliva kit. During the Facebook campaign, 338 people with ASD were enrolled, of whom 312 (92.3%) consented to providing a saliva sample and 167 (49.4%) returned the saliva kit (see Tables 2 and 3).

**Table 2:** Number of participants enrolled during and between the media campaigns.

Participants	Radio campaign (11/28/2016-01/31/2017)	Time between campaigns (02/01/2017-03/22/2017)	Social media campaign (03/23/2017-05/23/2017)	Total sample (03/01/2016-12/31/2018)
Enrolled, n	149	81	338	2051
Consented to DNA test, n (%)	140 (94.0)	68 (84.0)	312 (92.3)	1925 (93.9)
Returned saliva kit, n (%)	83 (55.7)	45 (55.6)	167 (49.4)	1126 (54.9)

**Table 3:** Race and ethnicity of participants from media campaigns compared to the population of Texas.

Characteristic		Radio campaign (11/28/2016-01/31/2017; n=149), n (%)	Time between campaigns (02/01/2017-03/22/2017; n=81), n (%)	Social media campaign (03/23/2017-05/23/2017; n=338), n (%)	Total sample (03/01/2016-12/31/2018; n=2051), n (%)	Texas, % [26]
**Race**						
	White	84 (75.0)	48 (75.0)	191 (74.9)	1155 (73.4)	78.8
	Black or African American	12 (10.7)	5 (7.8)	33 (12.9)	198 (12.6)	12.8
	Asian or Pacific Islander	8 (7.1)	4 (6.3)	6 (2.4)	73 (4.6)	5.3
	Native American or American Indian	0 (0.0)	0 (0.0)	0 (0.0)	4 (0.3)	1.0
	More than one race	5 (4.5)	4 (6.3)	18 (7.1)	92 (5.8)	2.0
	Other	3 (2.7)	3 (4.7)	7 (2.7)	52 (3.3)	N/A
	Data unavailable	37 (24.8)	17 (21.0)	83 (24.6)	477 (23.3)	N/A
**Ethnicity**						
	Hispanic	41 (35.0)	25 (39.7)	73 (28.5)	546 (34.3)	39.4
	Non-Hispanic	76 (65.0)	38 (60.3)	183 (71.5)	1044 (65.7)	N/A
	Data unavailable	32 (21.5)	18 (22.2)	82 (24.3)	461 (22.5)	N/A

#### Questionnaire Respondents

Among the 374 participants who responded to the REDCap questionnaire, 139 (37.2%) reported seeing the study advertised via social media prior to registering. Among this group, 75/139 (54.0%) rated this form of contact as the most influential in their decision to enroll. In contrast, only 9/374 survey respondents (2.4%) reported hearing the advertisement for the study over the radio, and among this group, 6 (66.7%) said that this form of contact was the most influential.

### Cost

**Figure 1:**
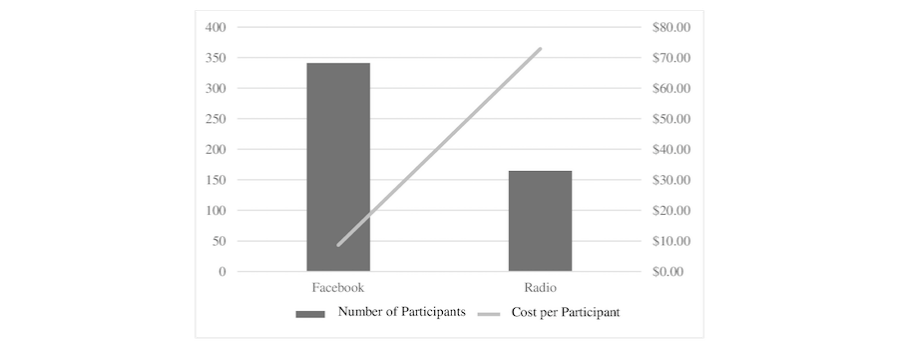
Comparison of the cost-efficiency of the Facebook and radio advertising campaigns.

As depicted in Figure 1, at an investment of $12,030 for radio, the cost per enrolled participant with ASD was $80.74 ($12,030/149). The cost of the Facebook campaign was $2950, for a per-participant cost of $8.73 ($2950/338).

### Geographic Reach

**Figure 2:**
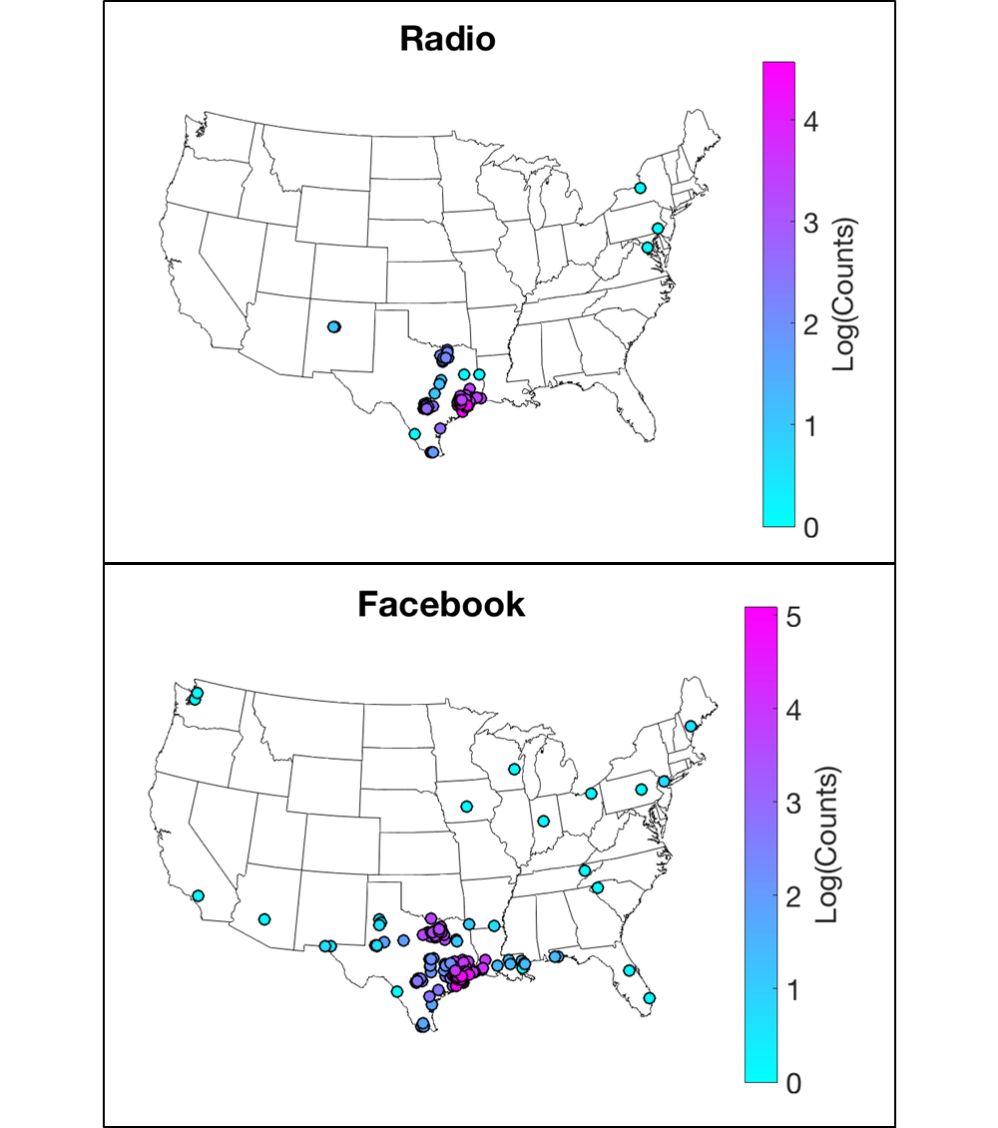
Comparison of the geographic reach of the radio and Facebook advertising campaigns.

Participants who enrolled in the SPARK project during the radio campaign were associated with 113 zip codes (Figure 2). These included the two states in which the radio campaign took place, plus four additional states. However, during the Facebook campaign, enrollment profiles were created from 208 zip codes across 16 states: the 2 states where the campaigns occurred, plus 14 additional states. Among participants who completed the REDCap questionnaire, those who identified social media as the most influential form of contact exhibited significant geographic differences compared to the rest of the sample. Of the individuals most influenced by social media, 13% indicated residence in a state outside of Texas, compared to only 5% of those most influenced by other strategies (n=367, χ^2^_1_=5.85, *P*=.02). There were no significant differences between these groups in terms of the likelihood to return saliva samples, racial or ethnic composition, or educational level. Only six participants endorsed radio advertisements as the most influential method of contact; this small subsample precluded comparable analyses for this recruitment method.

## Discussion

### Principal Results

In this study, we sought to determine the effectiveness of two paid advertising campaigns for recruitment in a web-based autism study. The results clearly demonstrated that paid advertising on social media substantially outperformed paid advertising on radio for the SPARK project. This was shown across the three examined parameters of numbers of enrolled participants, cost, and geographic reach. More than twice as many individuals with ASD were enrolled in the project during the social media campaign, and over half of the individuals who learned about SPARK via social media indicated that this form of contact was the most influential in their decision to participate. The Facebook campaign also proved to be more cost-effective; advertising on radio cost almost 10 times as much per enrolled participant as advertising on social media. Finally, the zip codes provided by the participants when they enrolled on the internet showed that social media had a much broader geographic reach than radio; participants enrolled via Facebook from 14 states *outside* the 2 states where the advertisements originated, compared to only 4 outside states for the radio campaign.

Several reasons could explain the success of Facebook advertising compared to radio advertising for recruiting SPARK participants. First, advertisements on social media can be tailored specifically to people who meet certain criteria. The advertisements used here were targeted to participants within a specified age range who had already endorsed having an interest in ASD or special needs. This allowed our study team to avoid wasting “paid impressions” on people who were not likely to be interested in participating, which was not possible with the radio advertising campaign.

Another likely reason that Facebook outperformed radio is its ability to expand the reach of an advertisement through a single click. Paid Facebook advertisements can be easily shared to additional profiles, pages, and groups, yielding many additional “organic impressions” for paid content at no extra cost. One Facebook share can reach hundreds or even thousands of other people depending on that person’s social circle and, if it is shared again, a virtual snowball phenomenon can emerge. This organic sharing process is also likely the reason we received so many registrations from states in which we did not advertise. Indeed, this particular advertisement received hundreds of shares and comments, and often the person sharing would “tag” people they thought would be interested in the advertisement, causing a personalized notification to be sent directly to that individual. In contrast, radio advertising is not easily shared and is certainly not shareable in real time. Furthermore, in radio advertising, a specific number of ad placements is agreed upon ahead of time by all parties, with no possibility of free “organic impressions.” Additionally, while both paid advertisements are technically temporary, when paid content is shared organically on Facebook, it becomes semipermanent. When the advertisement is shared to a person’s profile, page, or group, it will stay there until it is deleted or until newer content pushes it down the timeline feed. This allowed our Facebook advertisements to remain influential even after the advertising campaign technically ended (profiles created after the paid campaign period were omitted in the current study).

Finally, the success of the Facebook campaign may also be due to the social influence of social media. When listening to the radio, one cannot easily connect with other listeners. However, social networking sites allow individuals to instantly connect and communicate with other people anywhere in the world. Parents of children with special needs, including ASD, have been shown to frequently turn to other parents as sources of information and to heavily use online support groups, such as those on Facebook [18-20]. Indeed, our ad was shared in many online autism support groups, yielding additional organic impressions to a targeted audience who may have already been accustomed to receiving health-related advice and information from other parents in this way [18]. Further, studies have shown that our social networks can influence a wide range of behaviors, including offline behaviors such as exercise frequency [28]. Participation in a research study may thus also be influenced by a person in an individual’s social network sharing information about the study.

### Limitations

Although we were able to demonstrate the value of paid advertisements on social media for research recruitment, this study has a number of limitations. First, participants were not asked at the time of enrollment how they heard about the study; therefore, it is possible that participants who enrolled during the campaigns heard about SPARK through other mechanisms mentioned earlier, such as flyers, word of mouth, or the clinic website. However, these “background” recruitment efforts were consistent for both campaigns and thus were not expected to differentially impact the results. Furthermore, participants completed the REDCap survey up to one year post-enrollment, which may have affected the accuracy of the participants’ responses. However, the data from the questionnaire and the number of participants who enrolled during the campaigns are consistent with each other, with a clear advantage seen in social media recruitment in both cases.

### Conclusions

Despite the rising prevalence of ASD in the United States and our limited understanding of its causes, there is a surprising lack of studies examining how to recruit families with ASD into research studies. Because ASD is a heterogeneous condition both phenotypically and genetically, the sample size for etiological studies must be large, and efficiently recruiting participants will continue to be of primary importance. Understanding how to quickly enroll eligible participants allows research to progress more rapidly, bolstering the likelihood of success in understanding and treating ASD and related conditions. We concluded that participant recruitment via social media—specifically Facebook advertising—was superior to radio outreach across multiple parameters (participant numbers, cost, and geographic reach) for a web-based ASD-focused study that included submission of saliva samples for genetic analysis. Research teams attempting to target individuals with ASD should consider Facebook and other social media platforms as a viable, cost-effective recruitment strategy, including projects that have offline components (eg, clinical assessments and medical procedures).

Future efforts should examine whether the success in Facebook advertising described here can be replicated in other types of studies, such as those with wholly in-person procedures, and in different patient populations. Additionally, it will be important to similarly evaluate the success of other common recruitment strategies, such as printed materials (eg, brochures and fliers), community events, or provider referrals, across multiple parameters; also, it should be determined which combinations of recruitment strategies yield the greatest return on investment. Collectively, this type of research stands to inform best practices with regard to efficient, cost-effective recruitment strategies that ensure the successful completion of studies and subsequent advancement of scientific knowledge.

## Abbreviations

ASD: Autism Spectrum Disorder
REDCap: Research Electronic Data Capture
SFARI: Simons Foundation Autism Research Initiative
SPARK: Simons Foundation Powering Autism Research for Knowledge
